# Pressure‐dependent coupling between arterial pressure and intercostal muscle blood‐flow index during acute hypotension in humans

**DOI:** 10.1113/EP093687

**Published:** 2026-03-31

**Authors:** Masashi Ichinose, Mikie Nakabayashi, Yumie Ono

**Affiliations:** ^1^ Human Integrative Physiology Laboratory, School of Business Administration Meiji University Suginami‐ku Tokyo Japan; ^2^ Faculty of Engineering Niigata University Niigata Japan; ^3^ Institute for Research Administration Niigata University Niigata Japan; ^4^ Department of Electronics and Bioinformatics School of Science and Technology Meiji University Kanagawa Japan

**Keywords:** diffuse correlation spectroscopy, integrative haemodynamics, respiratory muscle blood flow

## Abstract

Regulation of respiratory muscle blood flow during acute systemic hypotension remains poorly understood in humans because continuous assessment of microvascular perfusion during rapid haemodynamic transients has been technically challenging. Using diffuse correlation spectroscopy, we investigated second‐by‐second coupling between mean arterial pressure (MAP) and intercostal muscle blood‐flow index (BFI) during an abrupt hypotensive stimulus in healthy humans. Fifteen young men underwent 5 min of suprasystolic bilateral thigh occlusion followed by rapid cuff release. The MAP and intercostal BFI were analysed at 1 Hz over drop (0–10 s) and recovery (10–20 s) phases. Phase‐specific MAP–BFI gain (%BFI per 1% change in MAP) was estimated using linear mixed‐effects models, with within‐participant centring. Immediately after cuff release, MAP and BFI decreased almost concomitantly, reached nadirs at ∼10 s and recovered towards baseline within the next ∼10 s. Phase‐averaged reductions in BFI were associated with reductions in MAP during the drop phase (Pearson's *r* = 0.66, *P* = 0.00701). Mixed‐effects modelling revealed near‐unity MAP–BFI gain in both phases (drop, 0.89 [95% confidence interval 0.63–1.15], *P* < 0.001 vs. 0, *P* = 0.417 vs. 1; recovery, 0.95 [0.50–1.40], *P* < 0.001 vs. 0, *P* = 0.830 vs. 1), with no phase difference (recovery–drop = 0.06 [−0.46 to 0.58], *P* = 0.824). Individual‐level gains showed greater variability, particularly during recovery. These findings demonstrate tight pressure–BFI coupling over seconds‐long time scales, indicating that perfusion pressure is a key determinant of intercostal microvascular blood‐flow dynamics during acute hypotensive stress.

## Abstract (highlights)

- **What is the central question of this study?**
  How does intercostal muscle blood‐flow index (BFI) respond to an abrupt fall in arterial pressure, and how tightly are second‐by‐second changes in intercostal BFI coupled to changes in mean arterial pressure in humans?

- **What is the main finding and its importance?**
  Using diffuse correlation spectroscopy, intercostal BFI closely tracked mean arterial pressure during acute hypotension, showing near‐unity pressure–BFI coupling during both the pressure drop and recovery. No significant associations were observed with heart rate or ventilatory variables. These findings identify perfusion pressure as a key determinant of intercostal microvascular haemodynamics.

## 1 INTRODUCTION

The respiratory muscles are essential skeletal muscles that must sustain aerobic work continuously; accordingly, the regulation of their blood flow and oxygen delivery is crucial for preserving contractile performance. Classic diaphragm experiments demonstrate that when the tension–time load exceeds a critical threshold, diaphragmatic blood flow becomes mechanically limited and endurance declines (Bark et al., 1987; Bellemare & Grassino, 1982; Bellemare et al., 1983). Systemic hypotension further restricts diaphragmatic perfusion, reinforcing the tight coupling between perfusion pressure and respiratory muscle function (Hussain et al., 1989). Beyond the diaphragm, human intercostal perfusion assessed over an intercostal space rises with ventilatory demand, yet, despite a matched work of breathing, can be constrained during maximal whole‐body exercise (Vogiatzis et al., 2009), consistent with competition for cardiac output. In addition, expiratory‐flow limitation during exercise augments intercostal perfusion while reducing intercostal oxygenation, indicating a supply–demand mismatch (Athanasopoulos et al., 2010; Guenette et al., 2008). Thus, understanding the circulatory regulation of respiratory muscles is fundamental to integrative physiology.

Previous studies have provided extensive insights into the mechanisms controlling blood flow to locomotor muscles during exercise and hypoperfusion. For example, the interplay among central haemodynamic regulation, sympathetic vasoconstrictor influences and local metabolic vasodilatation has been investigated primarily in the limbs (Joyner & Casey, 2015; Rowell, 1993; Saltin & Calbet, 2006). In contrast, the regulation of respiratory‐muscle blood flow, particularly its dynamic adjustments during rapid cardiovascular perturbations, remains largely unexplored. Although respiratory‐muscle metaboreflex activation has been shown to influence sympathetic outflow and vascular resistance in peripheral tissues (Sheel et al., 2001; St Croix et al., 2000), much less is known about how the perfusion of the respiratory muscles themselves is regulated when systemic blood pressure suddenly changes. Acute reductions in arterial pressure occur in both physiological and clinical contexts, including orthostatic stress, acute blood loss and perioperative haemodynamic instability. Because respiratory muscles must maintain ventilation even during haemodynamic challenge, understanding how their microvascular perfusion responds to abrupt pressure perturbations is essential for interpreting integrated cardiovascular–respiratory regulation under stress.

One reason for this limited understanding is methodological. Conventional tools, such as Doppler ultrasound, can track conduit flow but provide poor acoustic windows to small intercostal branches, do not isolate intramuscular microvascular perfusion and lack breath‐by‐breath temporal fidelity; catheter‐based flowmetry, although feasible in exceptional critical‐care settings, is highly invasive and not practical for routine human studies. Pioneering catheter‐based flowmetry by Aubier et al. (1989) (direct sampling of phrenic venous effluent in mechanically ventilated patients with chronic obstructive pulmonary disease) offered rare, direct measurements of diaphragmatic blood flow and demonstrated dopamine‐associated increases in perfusion and contractile strength, an important proof of concept; nevertheless, the invasive protocol and clinical context limit generalizability. Near‐infrared spectroscopy with Indocyanine Green (NIRS–ICG) has therefore been the principal approach to interrogate intercostal perfusion in vivo; however, because it relies on bolus tracking, it yields intermittent estimates and is not designed for uninterrupted, second‐to‐second monitoring during abrupt systemic pressure transients (Athanasopoulos et al., 2010; Guenette et al., 2008; Vogiatzis et al., 2009). More recently, contrast‐enhanced ultrasound has expanded the toolkit by quantifying relative diaphragm blood flow in humans with good reliability; nevertheless, protocols require intravenous contrast and brief breath‐holds, and acquisitions are typically short cine loops rather than continuous recordings (Bird et al., 2024). As a result, the short‐term responses of respiratory muscle blood flow to acute arterial pressure fluctuations have remained difficult to evaluate in humans.

Diffuse correlation spectroscopy (DCS) enables non‐invasive, tracer‐free assessment of microvascular blood flow dynamics in deep skeletal muscle with high temporal resolution (Durduran et al., 2010; Ichinose et al., 2021; Rodrigues et al., 2024). Applied over the intercostal region, DCS provides a means to characterize seconds‐scale changes in a blood‐flow index (BFI) elicited by abrupt perturbations in arterial pressure; a capability not readily attainable with bolus‐tracking or short cine‐loop approaches. In this study, we use this capability to link systemic haemodynamics to intercostal BFI. Notably, in our recent work we applied DCS to the human intercostal musculature for the first time and showed that limb‐muscle metaboreflex activation increased intercostal BFI via a systemic pressor response, with no evidence of local vasoconstriction, consistent with blood pressure‐dependent regulation of intercostal muscle perfusion (Ichinose et al., 2025).

Accordingly, we aimed to determine how intercostal BFI responds to a sudden fall in arterial blood pressure in healthy humans. We hypothesized that intercostal BFI would change in close association with mean arterial pressure (MAP), yielding an approximately proportional MAP–BFI coupling at a second‐by‐second time scale. By quantifying this short‐term pressure–BFI coupling, our study places intercostal microvascular haemodynamics within an integrative framework and provides insight into how abrupt hypotension might compromise the perfusion of muscles supporting ventilation.

## 2 MATERIALS AND METHODS

### 2.1 Ethical approval

The study was approved by the Institutional Review Board of the School of Science and Technology at Meiji University (approval number 22‐560). All participants provided written informed consent prior to participation. The study conformed to the standards set by the latest revision of the *Declaration of Helsinki*, except for registration in a database.

### 2.2 Participants

We studied 15 healthy male volunteers with a median age of 21 [interquartile range: 19–22] years, body mass of 65.0 [64.0–79.5] kg and height of 174 [169–175] cm. Participants were limited to males to minimize potential sex‐related confounding, including hormonal fluctuations across the menstrual cycle (Sudhir et al., 1995) and documented sex differences in sympathetic vasoconstrictor responsiveness (Hogarth et al., 2007). None of the participants was taking any medications, and all were non‐smokers. Participants were instructed to abstain from caffeine, alcohol and vigorous physical activity for ≥12 h prior to the experiment.

### 2.3 Procedures

All experiments were conducted in a temperature‐controlled room (∼25°C). Upon entering the laboratory, each participant adopted a semi‐recumbent position, with the legs extended and supported at the level of the hips. Rapidly inflatable cuffs for arterial occlusion were placed on both thighs. After instrumentation was completed, a minimum rest period of 15 min was allowed before initiating data collection.

The protocol began with a 2 min rest period, followed by inflation of bilateral thigh cuffs to suprasystolic pressure (>240 mmHg) for 5 min. Immediately after the 5 min occlusion, the cuffs were rapidly deflated, and measurements were continued for an additional 3 min. Thigh‐cuff release after suprasystolic occlusion is a classic, non‐pharmacological method to induce an abrupt, transient fall in arterial blood pressure without the need for vasoactive drug administration (Aaslid et al., 1989; Ichinose et al., 2013).

### 2.4 Measurements

Heart rate (HR) was monitored using a three‐lead ECG. Beat‐to‐beat changes in arterial blood pressure were assessed using finger photoplethysmography (Finometer; Finapres Medical Systems, The Netherlands); the monitoring cuff was placed around the middle finger of the left hand, with the forearm and hand supported to align the cuff at heart level. Before data collection, the system was calibrated to brachial artery pressure at rest using an automated oscillometric device, in accordance with the manufacturer's instructions. The pressure in the occlusion cuffs was measured using a pressure transducer. Analog signals representing the ECG, arterial pressure waveform and occlusion cuff pressure were digitized at a sampling frequency of 1 kHz using an analog‐to‐digital converter (NI DAQCard‐6062E, National Instruments, USA) and recorded on a personal computer. Beat‐to‐beat HR and MAP were calculated using an offline data analysis program.

Ventilatory variables, including minute ventilation (*V̇*_E_) and end‐tidal carbon dioxide pressure ($P_{\mathrm{ETC}O_{2}}$), were measured breath by breath using a face mask connected to an electric gas flow meter and gas analyser (AE‐300s, Minato Medical Science, Japan).

A DCS system developed in our laboratory was used to evaluate microvascular haemodynamics in the intercostal muscles. A detailed description of the system has been published previously (Ichinose et al., 2018; Matsushita et al., 2025; Nakabayashi & Ono, 2017). Briefly, the system includes a long‐coherence continuous‐wave laser (785 nm, 100 mW; DL785‐100‐S, CrystaLaser, USA) and a photon‐counting avalanche photodiode detector (SPCM‐AQRH‐14‐FC; Excelitas, USA). A custom‐made collimator was affixed to the emitter probe to ensure safe energy density at the skin surface. Autocorrelation analysis of the detected light intensity time course and BFI estimation followed the methods described by Durduran et al. (2010) and Dong et al. (2012). The DCS system detects fluctuations in reflected near‐infrared light intensity caused by moving red blood cells, from which BFI is derived. Light intensity data were collected at 400 kHz using a LabVIEW‐based acquisition system (v.2022; National Instruments, USA), and BFI was computed at 1 s intervals. The source–detector separation was 3 cm, corresponding to an estimated effective sampling depth of ∼1.5 cm (Nakabayashi et al., 2023; Patterson et al., 1995). The probes were mounted in a fixed rubber holder and attached to the skin with medical adhesive tape at the midaxillary line of the seventh left intercostal space. The position of the intercostal muscle was identified by ultrasound imaging prior to probe placement, and the probes were then affixed accordingly (iU22; Philips, The Netherlands).

Beat‐to‐beat HR and MAP, and breath‐by‐breath *V̇*_E_ and $P_{\mathrm{ETC}O_{2}}$, were interpolated and resampled to 1 Hz to match the BFI time base. To isolate the temporal relationship between BFI and arterial pressure better, we applied a four‐point (4 s) moving average to attenuate high‐frequency respiratory fluctuations; the smoothed series were used for all subsequent analyses and figures.

In a separate session at rest, the thickness of the near‐surface tissue layers (skin and adipose) at the DCS measurement site was assessed using B‐mode ultrasound with a 17 MHz linear array transducer. The thickness of the near‐surface layers at the DCS measurement sites was 4.9 [4.2–5.6] mm.

### 2.5 Data analysis

Values of haemodynamic and respiratory variables at rest were calculated by averaging the data obtained during the 2 min rest period. To evaluate temporal responses to abrupt hypotension induced by thigh‐cuff release, for each participant, values were converted to percentage change from baseline using the 30 s pre‐release window as the reference. Both arterial blood pressure and BFI dropped sharply immediately after cuff release, reaching their lowest values in ∼10 s, then recovered to baseline within another 10 s. Therefore, the 20 s period immediately after cuff release was designated for analysis, with the following analysis intervals set relative to cuff release: 0–20 s (overall), 0–10 s (drop) and 10–20 s (recovery).

### 2.6 Phase‐averaged changes and correlations

We computed phase‐averaged percentage changes from baseline for each variable across the overall 0–20 s period and separately for the drop (0–10 s) and recovery (10–20 s) phases. To assess covariation with intercostal BFI, we correlated, within drop and recovery phases, the participant‐level mean changes in %BFI with the corresponding mean changes in %MAP, %HR, %*V̇*_E_ and $\%P_{\mathrm{ETC}O_{2}}$.

### 2.7 Evaluation of the correlation between second‐by‐second changes in MAP and intercostal BFI

#### 2.7.1 Primary group‐level gain (phase‐specific linear mixed‐effects model)

Within each phase (p = drop or recovery) using the second‐by‐second (1 Hz) data, the coupling (‘gain’) between MAP and BFI was quantified as the change in %BFI per 1% change in MAP (expressed as percentage change from baseline) using a linear mixed‐effects model (Gelman & Hill, 2007; Pinheiro & Bates, 2000). To isolate within‐participant covariation, %MAP was centred within each participant and phase. Specifically, for participant j at time t in phase p, the model was specified as:

$${\%\mathrm{BFI}}_{j,t}^{\left(p\right)}=\beta_{0}^{\left(p\right)}+\beta_{1}^{\left(p\right)}\left({\%\mathrm{MAP}}_{j,t}^{\left(p\right)}-\;{\bar{\%\mathrm{MAP}}}_{j}^{\left(p\right)}\right)+b_{0j}^{\left(p\right)}$$

$$+b_{1j}^{\left(p\right)}\left(\%{\mathrm{MAP}}_{j,t}^{\left(p\right)}-\;{\bar{\%\mathrm{MAP}}}_{j}^{\left(p\right)}\right)+\varepsilon_{j,t}^{\left(p\right)}.$$

Here, $\beta_{0}^{\left(p\right)}$ and $\beta_{1}^{\left(p\right)}$ denote the phase‐specific fixed intercept and slope, respectively, while $b_{0j}^{\left(p\right)}$ and $b_{1j}^{\left(p\right)}$ represent participant‐specific random intercepts and slopes; ${\bar{\%MAP}}_{j}^{\left(p\right)}$ is the mean %MAP for participant j within phase p; and $\varepsilon_{j,t}^{\left(p\right)}$ is the residual error. Models were estimated using restricted maximum likelihood (REML). For each phase, we report the fixed‐effect slope $\beta_{1}^{\left(p\right)}$ with 95% confidence intervals (CIs). To compare phase‐specific gains, we computed the between‐phase difference $\left({\hat{\beta }}_{1}^{\left(\mathrm{recovery}\right)}-{\hat{\beta }}_{1}^{\left(\mathrm{drop}\right)}\right)$ using the phase‐specific slope estimates and their standard errors. The standard error of the contrast was obtained from the two phase‐specific standard errors assuming zero covariance, and degrees of freedom were approximated using the Welch–Satterthwaite method.

### 2.8 Participant‐level slopes

Independently of the mixed‐effects estimation, within each participant and phase we fitted an ordinary least‐squares (OLS) regression of %BFI on %MAP using the same second‐by‐second data, yielding individual gains (slopes). Uncertainty for these individual gains is summarized with heteroscedasticity‐ and autocorrelation‐consistent (HAC) standard errors (Newey–West, lag = 3) and corresponding 95% CIs.

### 2.9 Statistical analysis

Data are presented as medians with interquartile ranges, unless otherwise specified. Normality was assessed using the Shapiro–Wilk test with visual inspection (histograms/Q–Q plots), and Student's paired *t*‐tests were used when normality assumptions were satisfied; otherwise, Wilcoxon signed‐rank tests were applied. Rest (2 min pre‐occlusion) versus pre‐release baseline (30 s) was evaluated on paired differences (baseline − rest). BFI was tested with the Wilcoxon signed‐rank test, whereas MAP, HR, *V̇*_E_ and $P_{\mathrm{ETC}O_{2}}$ were tested with Student's paired *t*‐tests. Phase‐averaged percentage changes from baseline were tested against 0% (i.e., baseline) for each post‐release interval (0–20, 0–10 and 10–20 s). Student's paired *t*‐tests were used for BFI, *V̇*_E_ and $P_{\mathrm{ETC}O_{2}}$; for MAP, Student's paired *t*‐tests were used for 0–20 and 0–10 s, and the Wilcoxon signed‐rank test was used for 10–20 s; for HR, Student's paired *t*‐tests were used for 0–20 and 10–20 s, and the Wilcoxon signed‐rank test was used for 0–10 s. To account for multiple comparisons within each variable, *P*‐values were adjusted using the Holm method. Associations between phase‐averaged percentage changes from baseline in BFI and corresponding changes in MAP, HR, *V̇*_E_ and $P_{\mathrm{ETC}O_{2}}$ were assessed separately for drop and recovery using Pearson's correlation coefficient as the primary measure of association; Spearman's rank correlation coefficient was also calculated and reported as a sensitivity analysis. For each phase, inference on the group gain (fixed‐effect slope of %BFI on %MAP from the mixed‐effects model defined in section 2.5 Data analysis) used Wald *t*‐tests for: (1) slope = 0; and (2) slope = 1, with corresponding Wald 95% CIs. Participant‐level slopes were tested against zero and one, and the false discovery rate (FDR) was controlled within phase and within each family of tests (vs. 0; vs. 1) using the Benjamini–Hochberg procedure. All hypothesis tests were two sided, and statistical significance was defined as *P* < 0.05. Statistical analyses were performed in R (v.4.5.0; R Foundation for Statistical Computing, Vienna, Austria) with the packages nlme, sandwich and lmtest.

## 3 RESULTS

### 3.1 Group mean time‐series changes across the occlusion–release protocol

**FIGURE 1:**
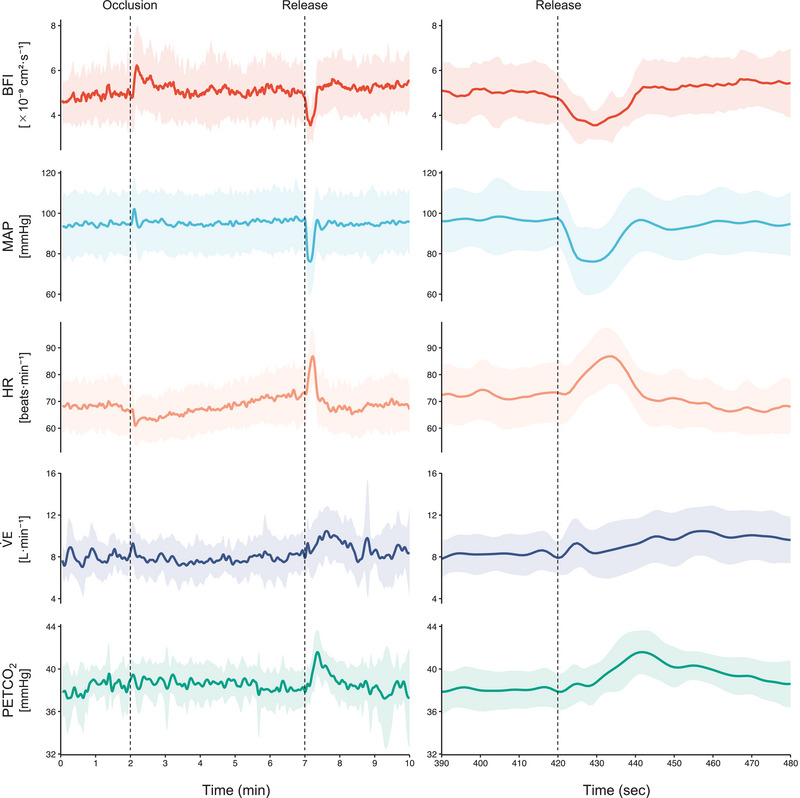
Group mean time‐series changes in cardiovascular and respiratory variables across the occlusion–release protocol. Left‐column panels show the entire protocol; right‐column panels show the window from 30 s before to 60 s after cuff release. Lines indicate group means, and shaded bands indicate the SD across participants (resampled at 1 Hz). Rest and pre‐release baseline summary values (medians and interquartile ranges) are provided in Table 1. After a 2 min resting measurement, both thighs were occluded for 5 min, followed by cuff release. Traces are displayed after a 4 s moving average to attenuate respiratory‐frequency fluctuations; the smoothed series were also used for all analyses (see Materials and Methods). Abbreviations: BFI, blood‐flow index; HR, heart rate; MAP, mean arterial pressure; $P_{\mathrm{ETC}O_{2}}$, end‐tidal carbon dioxide pressure; *V̇*_E_, minute ventilation. *n* = 15 participants.

**FIGURE 2:**
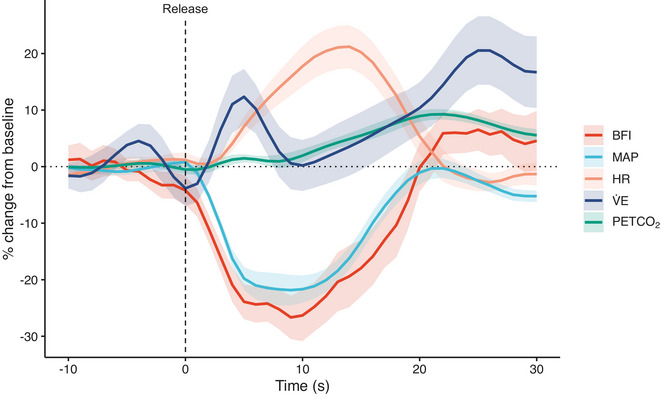
Percentage changes in cardiovascular and respiratory variables immediately after cuff release. The graph shows data from 10 s before to 30 s after cuff release. Values are expressed as the percentage change from the 30 s baseline immediately before release. Lines indicate group means, and shaded bands indicate the SEM across participants (resampled at 1 Hz) to facilitate visual comparison of temporal response patterns across variables. Traces are displayed after a 4 s moving average to attenuate respiratory‐frequency fluctuations; the smoothed series were also used for all analyses (see Materials and Methods). Interindividual variability of the baseline‐normalized phase responses is shown in Figure 3 (boxplots with individual data points). Abbreviations: BFI, blood‐flow index; HR, heart rate; MAP, mean arterial pressure; $P_{\mathrm{ETC}O_{2}}$, end‐tidal carbon dioxide pressure; *V̇*_E_, minute ventilation. *n* = 15 participants.

Figure 1 shows the group mean time‐series changes in cardiovascular and respiratory variables across the entire protocol, and Table 1 summarizes values at rest (2 min before occlusion) and during the 30 s baseline immediately before cuff release. Figure 2 depicts the same variables as the percentage change from baseline, from 10 s before to 30 s after release. Compared with rest, MAP (*P* = 0.0184) and HR (*P* = 0.00698) were slightly but significantly higher at the pre‐release baseline, whereas BFI (*P* = 0.580), *V̇*_E_ (*P* = 0.222) and $P_{\mathrm{ETC}O_{2}}$ (*P* = 0.629) did not differ significantly (Table 1). Although the medians were similar, the statistical significance for MAP reflects a small but consistent within‐participant increase from rest to the pre‐release baseline (means ± SD: rest, 94.5 ± 15.4 mmHg; baseline, 97.0 ± 15.1 mmHg). Immediately after cuff release, MAP and BFI fell almost concomitantly, reached nadirs at ∼10 s, then recovered towards baseline over the subsequent ∼10 s (Figures 1 and 2). HR rose rapidly, consistent with baroreflex‐mediated tachycardia in response to the abrupt hypotension, whereas *V̇*_E_ and $P_{\mathrm{ETC}O_{2}}$ increased more slowly over the post‐release period (Figures 1 and 2).

**TABLE 1:** Cardiovascular and respiratory variables at rest and during the pre‐release baseline.

Variable	Rest	Baseline	*P*‐value
BFI, ×10^−^^9^ cm^2^ s^−^^1^	4.51 (4.06–5.21)	4.78 (3.99–5.75)	0.580
MAP, mmHg	95.2 (86.5–100.9)	95.3 (86.7–104.0)	0.0184
HR, beats min^−^^1^	69.0 (61.8–73.4)	74.4 (64.1–80.3)	0.00698
*V̇*_E_, L min^−^^1^	7.7 (7.3–8.7)	8.1 (7.3–8.9)	0.222
$P_{\mathrm{ETC}O_{2}}$, mmHg	38.2 (37.3–39.2)	38.1 (37.1–38.5)	0.629

*Note*: Values are medians (interquartile ranges). The *P*‐values are for paired comparisons between rest (2 min before occlusion) and baseline (final 30 s before cuff release). Abbreviations: BFI, blood‐flow index; HR, heart rate; MAP, mean arterial pressure; $P_{\mathrm{ETC}O_{2}}$, end‐tidal carbon dioxide pressure; *V̇*_E_, minute ventilation. Statistical comparisons were performed using the Wilcoxon signed‐rank test for BFI and Student's paired *t*‐tests for MAP, HR, *V̇*_E_ and $P_{\mathrm{ETC}O_{2}}$. *n* = 15 participants.

### 3.2 Phase‐averaged percentage changes from baseline

**FIGURE 3:**
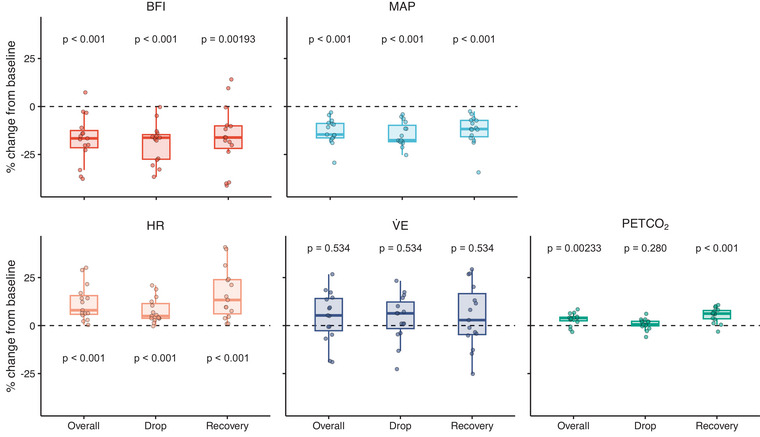
Phase‐averaged percentage changes from baseline in cardiovascular and respiratory variables. Participant‐level mean percentage changes in blood‐flow index (BFI), mean arterial pressure (MAP), heart rate (HR), minute ventilation (*V̇*_E_) and end‐tidal carbon dioxide pressure ($P_{\mathrm{ETC}O_{2}}$) are shown for the overall (0–20 s), drop (0–10 s) and recovery (10–20 s) phases following occlusion release. Points represent individual participants (*n* = 15). Box plots indicate the median and interquartile range; whiskers extend to 1.5 times the interquartile range. The horizontal dashed line denotes baseline (0%). Statistical comparisons between each phase and baseline were performed using Student's paired *t*‐tests, except for MAP in recovery and HR in drop, which were analysed using Wilcoxon signed‐rank tests. Holm‐adjusted *P*‐values are shown for each phase.

Figure 3 shows participant‐level percentage changes in BFI, MAP, HR, *V̇*_E_ and $P_{\mathrm{ETC}O_{2}}$ averaged over the overall, drop and recovery phases relative to baseline. BFI and MAP were significantly reduced in all phases (BFI: overall and drop, *P* < 0.001, recovery, *P* = 0.00193; MAP: overall, drop and recovery, *P* < 0.001), whereas HR was significantly increased in all phases (overall, drop and recovery, *P* < 0.001). $P_{\mathrm{ETC}O_{2}}$ increased significantly during the overall (*P* = 0.00233) and recovery (*P* < 0.001) phases, but not during the drop phase (*P* = 0.280). No significant changes were observed in *V̇*_E_ in any phase (all *P* = 0.534).

### 3.3 Phase‐averaged associations between changes in intercostal BFI and cardiovascular and respiratory variables

**FIGURE 4:**
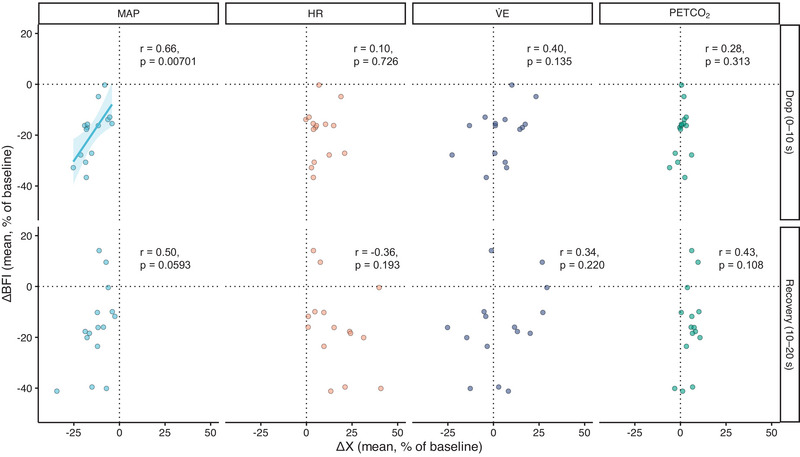
Phase‐averaged associations between changes in blood‐flow index (BFI) and cardiovascular and respiratory variables. Scatter plots show the relationships between phase‐averaged percentage changes from baseline in BFI and corresponding changes in mean arterial pressure (MAP), heart rate (HR), minute ventilation (*V̇*_E_) and end‐tidal carbon dioxide pressure ($P_{\mathrm{ETC}O_{2}}$) during the drop (0–10 s) and recovery (10–20 s) phases following occlusion release. ΔX represents the participant‐level mean percentage change from baseline for each variable averaged over the corresponding phase. Each point represents an individual participant (*n* = 15). A linear regression line with 95% confidence interval is shown only for associations that were statistically significant by Pearson's correlation. Correlations were quantified using Pearson's correlation coefficient, with Spearman's rank correlation coefficient reported as a sensitivity analysis.

Figure 4 illustrates the relationships between phase‐averaged percentage changes in BFI and corresponding changes in cardiovascular and respiratory variables during the drop and recovery phases following occlusion release. During the drop phase, changes in %BFI were positively associated with changes in %MAP (Pearson's *r* = 0.66, *P* = 0.00701; Spearman's ρ = 0.79, *P* < 0.001). In contrast, no significant associations were observed between changes in %BFI and changes in %HR (Pearson's *r* = 0.10, *P* = 0.726; Spearman's ρ = 0.05, *P* = 0.860), %*V̇*_E_ (Pearson's *r* = 0.40, *P* = 0.135; Spearman's ρ = 0.26, *P* = 0.348) or $\%P_{\mathrm{ETC}O_{2}}$ (Pearson's *r* = 0.28, *P* = 0.313; Spearman's ρ = 0.31, *P* = 0.265) during this phase. During the recovery phase, changes in %BFI were not significantly correlated with changes in %MAP when assessed using Pearson's correlation (*r* = 0.50, *P* = 0.0593), whereas Spearman's rank correlation indicated a significant monotonic association (ρ = 0.56, *P* = 0.0297). No significant correlations were detected between changes in %BFI and changes in %HR (Pearson's *r* = −0.36, *P* = 0.193; Spearman's ρ = −0.49, *P* = 0.0620), %*V̇*_E_ (Pearson's *r* = 0.34, *P* = 0.220; Spearman's ρ = 0.34, *P* = 0.221) or %$P_{\mathrm{ETC}O_{2}}$ (Pearson's *r* = 0.43, *P* = 0.108; Spearman's ρ = 0.24, *P* = 0.383) during the recovery phase.

### 3.4 Per‐phase MAP–BFI gain estimated from second‐by‐second data

**FIGURE 5:**
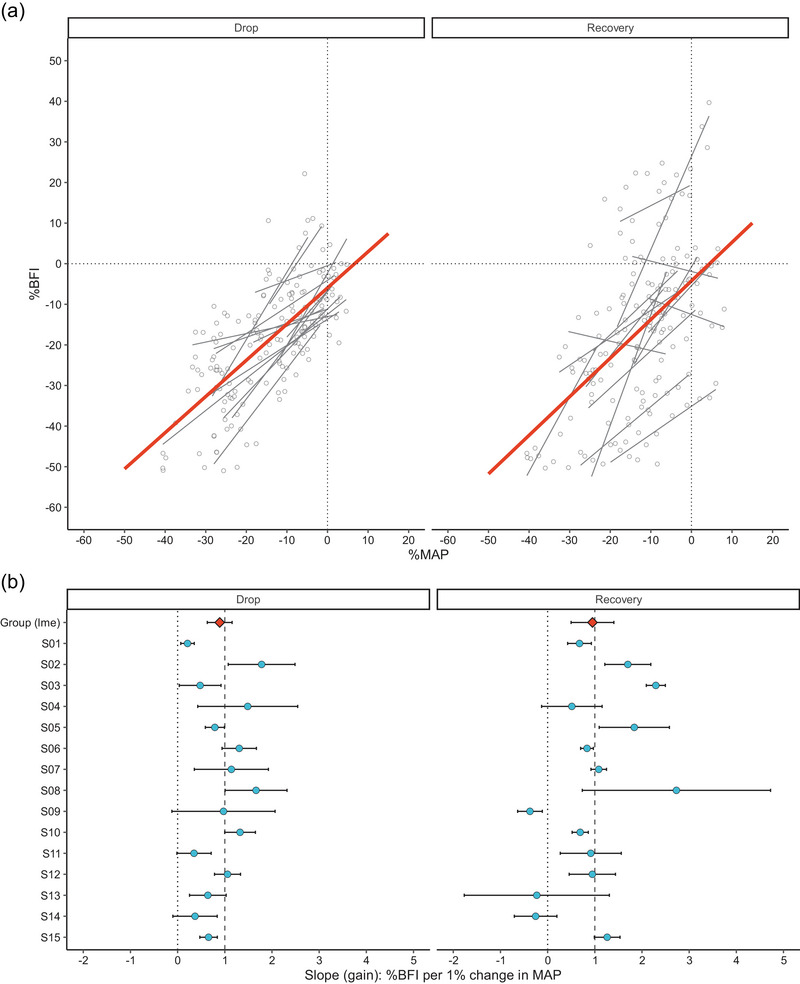
Phase‐specific gain between mean arterial pressure (MAP) and blood‐flow index (BFI) estimated from second‐by‐second data. (a) Scatter plot with model fits during drop (0–10 s; left) and recovery (10–20 s; right). Second‐by‐second %BFI is plotted against %MAP within each phase. Thin lines show participant‐wise ordinary least‐squares fits (points are second‐by‐second observations). The red continuous line is the phase‐specific group fit from a linear mixed‐effects model (REML) with within‐participant‐centred %MAP and random intercepts and slopes by participant; the line is plotted using the model‐implied intercept and slope for each phase. *n* = 15 participants. (b) Forest plot of gains (slopes) in drop (left) and recovery (right). Participant‐wise slopes (gain: change in %BFI per 1% change in MAP) are shown with 95% confidence intervals computed using heteroscedasticity‐ and autocorrelation‐consistent (Newey–West, lag = 3) standard errors. The top row, ‘Group (lme)’, displays the phase‐specific group slope and Wald 95% confidence intervals from the same mixed‐effects model as in panel (a), marked by a red diamond. Vertical reference lines indicate gain = 0 (no association) and gain = 1 (1:1 change). *n* = 15 participants.

Figure 5a shows second‐by‐second relationships between %MAP and %BFI in each phase, with thin per‐participant OLS lines and the mixed‐effects group line. Figure 5b summarizes per‐participant slopes with HAC 95% CIs and the group slope with Wald 95% CIs. Using second‐by‐second data, the group‐level gain estimated by a mixed‐effects model with within‐participant centring and random slopes was close to unity in both phases: 0.89 [95% CI 0.63–1.15] for drop and 0.95 [0.50–1.40] for recovery (Figure 5b). Both gains were clearly greater than zero (drop, *P* < 0.001; recovery, *P* < 0.001), did not differ from one (drop, *P* = 0.417; recovery, *P* = 0.830) and showed no evidence of a phase difference (recovery−drop = 0.06 [−0.46–0.58], *P* = 0.824). Thus, at the group level, the estimated MAP–BFI gain was near unity in both phases.

At the individual level, most participants showed a significant MAP–BFI association (slope ≠ 0 tests, FDR‐adjusted: 11/15 in drop and 12/15 in recovery). During the drop phase, slopes were significant for all participants except S03, S09, S11 and S14, and all significant slopes were positive (Figure 5b). During the recovery phase, slopes were significant for all participants except S04, S13 and S14; among those significant, 11 of 12 were positive, and one participant (S09) showed a significant negative slope. Unity tests against a slope of one (FDR‐adjusted) identified significant deviations in 4 of 15 participants during drop and in 7 of 15 during recovery. During drop, all significant deviations were positive but less than one (S01, S11, S14 and S15; Figure 5b). During recovery, deviations were more heterogeneous, with three of seven positive but less than one (S01, S06 and S10), two of seven greater than one (S02 and S03) and two of seven negative (S09 and S14). Overall, these participant‐level estimates and CIs indicated appreciable interindividual variability, whereas the phase‐specific mixed‐effects slopes remained close to unity (Figure 5). Complete participant‐level slope estimates, corresponding 95% CIs, and FDR‐adjusted *P*‐values for tests against zero and one are provided in Appendix Table A1.

## 4 DISCUSSION

This study is the first to quantify second‐by‐second coupling between MAP and intercostal BFI during an abrupt hypotensive transient in healthy humans using DCS. The principal finding is that intercostal BFI closely tracked changes in MAP during both the rapid drop and the subsequent recovery phases following thigh‐cuff release, with group‐level MAP–BFI gains near unity. These observations indicate that, over this short time scale, intercostal BFI is tightly coupled to systemic haemodynamics, consistent with largely pressure‐dependent behaviour during acute hypotensive stress.

A notable contribution of the present work is that it provides a continuous, second‐by‐second view of how intercostal BFI responds during an abrupt systemic pressure transient, which is an aspect of respiratory muscle haemodynamics that has remained largely inaccessible in humans using conventional approaches. By capturing these rapid dynamics, our results highlight the importance of temporal scale when interpreting vascular control. Specifically, during seconds‐long haemodynamic transients, the intercostal BFI response appears to exhibit limited buffering, such that reductions in arterial pressure are transmitted rapidly to the microvasculature. This pattern does not preclude slower regulatory influences, including sympathetic vasoconstrictor adjustments, endothelium‐dependent vasodilatation and the progressive emergence of local metabolic vasodilator signals as described for limb skeletal muscle (Joyner & Casey, 2015; Rowell, 1993; Saltin & Calbet, 2006), but suggests that pressure–flow coupling predominates over the early time window before local mechanisms can exert meaningful influence on intercostal muscle microvascular perfusion. This pressure–BFI coupling is consistent with a recent report from our group (Ichinose et al., 2025), in which intercostal BFI increased during limb‐muscle metaboreflex activation in concert with a systemic pressor response. In that study, we examined pressure augmentation during reflex activation, whereas the present work characterizes second‐by‐second pressure–BFI coupling during an abrupt hypotensive transient. Together, these findings support the view that arterial pressure is an important determinant of intercostal microvascular flow dynamics across distinct physiological contexts.

To probe pressure–flow coupling specifically on a seconds‐long time scale, we used the bilateral thigh‐cuff release model, which elicits a rapid, transient hypotensive stimulus without pharmacological intervention. Pharmacological approaches (e.g., titrated vasodilators) can provide complementary information, but they reduce arterial pressure primarily by directly altering vascular tone and might introduce additional reflex and regional conductance changes that complicate interpretation of second‐by‐second pressure–flow dynamics.

Separating the response into drop and recovery phases provided further insight into the dynamic nature of this coupling. During the initial drop phase, intercostal BFI decreased almost concomitantly with MAP (Figures 1 and 2), consistent with an immediate, pressure‐driven response. During recovery, although MAP and intercostal BFI again rose in parallel at the group level, greater interindividual variability in the estimated gains emerged (Figure 5). This heterogeneity during recovery might reflect the superimposition of multiple processes, including baroreflex‐mediated adjustments in cardiac output, redistribution of blood flow and evolving local vascular responses that influence intercostal muscle microvascular perfusion. Although reactive hyperaemia in the lower limbs might contribute to transient redistribution of cardiac output following cuff release, the close association between the magnitude of MAP change and intercostal BFI suggests that perfusion pressure, rather than redistribution per se, was the predominant determinant of the observed response. Nevertheless, despite this variability, the absence of a significant phase difference in group‐average gain underscores the robustness of pressure–BFI coupling across both phases of the transient.

Phase‐averaged analyses further supported a predominant association between changes in intercostal BFI and the magnitude of arterial pressure reduction. Larger decreases in MAP were accompanied by greater reductions in intercostal BFI, whereas no significant associations were observed between BFI and changes in HR, *V̇*_E_ or $P_{\mathrm{ETC}O_{2}}$ (Figure 4). Together with the second‐by‐second gain analyses, these findings indicate that the intercostal BFI response to acute hypotension is primarily related to the extent of the pressure perturbation rather than concurrent changes in ventilatory drive or reflex tachycardia.

The individual‐level analyses further emphasized the integrated and variable nature of intercostal haemodynamics. Although most participants demonstrated positive MAP–BFI coupling, the direction and magnitude of deviations from unity differed across individuals, particularly during recovery (Figure 5a, b). Such variability is likely to reflect differences in breathing pattern and chest wall mechanics, intrathoracic pressure fluctuations, autonomic responsiveness and vascular properties, all of which might influence the transmission of systemic pressure changes to the intercostal microvasculature. Importantly, the present study was not designed to disentangle these contributors, and the observed variability should be viewed as an inherent feature of an integrated physiological system rather than evidence for distinct regulatory phenotypes.

The present findings extend and complement prior work on respiratory muscle perfusion. Classic animal studies demonstrated that diaphragmatic blood flow becomes constrained when mechanical or haemodynamic limits are exceeded (Bark et al., 1987; Bellemare et al., 1983) and that systemic hypotension further compromises perfusion (Hussain et al., 1989). In humans, studies using NIRS–ICG indicate that intercostal perfusion rises with increasing ventilatory demand. However, even when the work of breathing is matched, intercostal perfusion can be blunted during maximal whole‐body exercise (Vogiatzis et al., 2009). In addition, expiratory‐flow limitation during exercise has been associated with increased intercostal perfusion accompanied by reduced intercostal oxygenation, consistent with a supply–demand mismatch (Athanasopoulos et al., 2010; Guenette et al., 2008). However, because NIRS–ICG relies on bolus tracking, it yields intermittent estimates and is not suited to uninterrupted, second‐by‐second monitoring during abrupt systemic pressure transients. In contrast, the present study leveraged the high temporal resolution of DCS to integrate systemic haemodynamics with intercostal BFI on a continuous time base, thereby providing a dynamic view of intercostal haemodynamics that is not readily attainable with bolus‐tracking approaches.

From a broader perspective, this work illustrates the value of an integrative haemodynamic approach to understanding the human vasculature system. Rather than focusing on a single biomarker or vascular bed in isolation, simultaneous consideration of systemic pressure and local microvascular flow dynamics revealed how closely intercostal BFI is embedded within whole‐body haemodynamic control during acute stress. Such integration aligns with emerging calls to adopt systems‐based experimental strategies that better capture the coordinated behaviour of the cardiovascular system across organs and time scales. Clinically, these findings suggest that during conditions characterized by abrupt or labile hypotension (e.g., orthostatic stress, acute blood loss or perioperative haemodynamic instability), intercostal muscle microvascular perfusion might be particularly susceptible to reductions in arterial pressure over short time scales. Although the present study was conducted in healthy participants, this pressure–BFI coupling raises the possibility that maintaining perfusion pressure could help to preserve perfusion of respiratory muscles when haemodynamic reserve is limited, warranting future studies in patient populations.

### 4.1 Limitations

Several limitations should be acknowledged. First, DCS provides an index of microvascular blood flow based on red blood cell motion rather than a direct measure of absolute perfusion or oxygen delivery, hence inferences are necessarily relative. In the present study, we did not obtain concurrent NIRS‐derived measures of total haemoglobin concentration or deoxyhaemoglobin and therefore cannot determine whether the observed changes in intercostal BFI were accompanied by alterations in blood volume or oxygen extraction. Accordingly, our findings should be interpreted as reflecting pressure‐dependent changes in a microvascular flow index, rather than definitive changes in tissue oxygen delivery or metabolic regulation.

Second, the DCS‐derived BFI might include contributions from overlying tissues (e.g., skin and subcutaneous adipose tissue) in addition to the underlying intercostal muscle (Bartlett et al., 2023; Nakabayashi et al., 2023); therefore, BFI should be interpreted as reflecting microvascular flow dynamics within the measurement volume rather than being exclusively muscle specific. Because the thigh‐cuff release protocol is not expected to elicit large thermoregulatory shifts, major confounding changes in skin perfusion are unlikely; nonetheless, superficial contributions cannot be excluded fully.

Third, thigh‐cuff release provides a non‐pharmacological, transient hypotensive stimulus; however, the magnitude of the MAP decrease differs across individuals. This feature is advantageous for illustrating that larger pressure reductions tend to be accompanied by larger reductions in intercostal BFI at the phase‐averaged level (Figure 4). At the same time, unequal MAP excursions can affect the precision of second‐by‐second coupling estimates: when within‐phase MAP variation is limited, the %BFI–%MAP slope is less well identified and becomes more sensitive to residual physiological fluctuations, which might reduce the apparent strength of coupling in some participants. Accordingly, we report both participant‐wise OLS slopes and a phase‐specific mixed‐effects estimate of the group gain, the latter being well suited to quantifying the population‐average within‐participant relationship while accommodating heterogeneity in individual responses (Gelman & Hill, 2007; Pinheiro & Bates, 2000). Fourth, measurements were obtained over a single intercostal site and might not fully represent perfusion across other respiratory muscles. Fifth, the study was limited to young healthy men, and sex‐related differences or effects of ageing and disease cannot be inferred. Finally, thigh‐cuff release represents a specific model of acute hypotension, and responses might differ under other haemodynamic challenges. These considerations notwithstanding, the present approach is well suited to interrogating short‐term, integrated haemodynamic responses in humans.

## 5 CONCLUSION

In conclusion, intercostal BFI closely follows systemic arterial pressure during abrupt hypotensive stress, with near‐unity pressure–BFI coupling over seconds‐long time scales. By integrating continuous measures of systemic haemodynamics and local microvascular flow dynamics, this study places intercostal muscle microvascular perfusion within a broader framework of human vascular physiology and highlights the utility of integrative haemodynamic technologies for advancing our understanding of the human vascular system.

## AUTHOR CONTRIBUTIONS

Masashi Ichinose performed the experiments and prepared the figures. Masashi Ichinose, Mikie Nakabayashi and Yumie Ono conceived and designed the study, analysed the data, interpreted the experimental results, drafted, edited and revised the manuscript, approved the final version and agree to be accountable for all aspects of the work in ensuring that questions related to the accuracy or integrity of any part of the work are appropriately investigated and resolved. All persons designated as authors qualify for authorship, and all those who qualify for authorship are listed.

## CONFLICT OF INTEREST

None.

## Data Availability

Source data for this study are not publicly available owing to privacy or ethical restrictions. The data will be made available to verified researchers upon reasonable request by contacting the corresponding author.

## 1

**TABLE A1:** Participant‐level mean arterial pressure–blood‐flow index slope estimates during the drop (0–10 s) and recovery (10–20 s) phases.

Phase	Participant	Slope	95% CI	*P*‐value (slope ≠ 0)	*P*‐value (slope ≠ 1)
Drop	S01	0.21	0.07–0.35	0.0290	<0.001
	S02	1.78	1.07–2.49	0.00175	0.0769
	S03	0.48	0.03–0.92	0.0798	0.0613
	S04	1.49	0.43–2.55	0.0308	0.462
	S05	0.79	0.59–0.99	<0.001	0.0866
	S06	1.31	0.94–1.67	<0.001	0.137
	S07	1.14	0.35–1.92	0.0290	0.781
	S08	1.66	1.01–2.32	0.00175	0.0866
	S09	0.97	−0.12 to 2.07	0.124	0.960
	S10	1.32	1.00–1.65	<0.001	0.0866
	S11	0.35	−0.02 to 0.71	0.108	0.00216
	S12	1.06	0.78–1.33	<0.001	0.781
	S13	0.64	0.25–1.03	0.0194	0.105
	S14	0.37	−0.10 to 0.84	0.158	0.0320
	S15	0.66	0.47–0.84	<0.001	0.00211
Recovery	S01	0.68	0.42–0.93	0.00111	0.0287
	S02	1.70	1.21–2.19	<0.001	0.0147
	S03	2.29	2.09–2.50	<0.001	<0.001
	S04	0.51	−0.13 to 1.15	0.175	0.171
	S05	1.84	1.09–2.58	0.00173	0.0516
	S06	0.83	0.70–0.97	<0.001	0.0358
	S07	1.08	0.92–1.25	<0.001	0.368
	S08	2.73	0.73–4.73	0.0315	0.134
	S09	−0.37	−0.64 to −0.11	0.0295	<0.001
	S10	0.69	0.52–0.86	<0.001	0.00153
	S11	0.91	0.27–1.56	0.0295	0.834
	S12	0.95	0.46–1.44	0.00721	0.834
	S13	−0.23	−1.77 to 1.31	0.776	0.160
	S14	−0.25	−0.71 to 0.20	0.320	<0.001
	S15	1.26	0.99–1.53	<0.001	0.0983

*Note*: Slopes were estimated using ordinary least squares with Newey–West heteroscedasticity‐ and autocorrelation‐consistent (HAC) standard errors. The *P*‐values are false discovery rate‐adjusted within phase and within each family of tests (slope ≠ 0 and slope ≠ 1) using the Benjamini–Hochberg procedure. Slope represents the change in percentage blood‐flow index per 1% change in mean arterial pressure. *n* = 15 participants.
